# Supplementary material for: Epidemiology and Transmitted HIV-1 Drug Resistance among Treatment-Naïve Individuals in Israel, 2010–2018
Source: Viruses. 2021 Dec 31;14(1):71. doi: 10.3390/v14010071 (PMC8779053; doi:10.3390/v14010071)
Supplement: Supplementary file 1 [file viruses-14-00071-s001.zip › viruses-1535594-supplementary.pdf]

Supplementary Material

Supplementary Methods section:

Detailed phylogenetic and statistical analysis:

Combined partial PR and RT sequence stripped of drug resistance codons were aligned with early reference sequences from the Los-Alamos database (https:www.HIV-1.lanl.gov) using MEGAX (version10.1.1) (1). General time-reversible model, with the proportion of invariable sites and gamma plus invariant sites-distributed rate heterogeneity (GTR+G+I model), was used based on the results of JModelTest (2) version 2.1.9. Maximum likelihood phylogenetic trees were constructed with branch support assessed from 1000 bootstrap replicates and visualized with Fig Tree version 1.4.4 (http://tree.bio.ed.ac.uk/software/figtree/). Only clusters of epidemiologic relevance (with posterior probability>0.7) were studied. Cluster validation was done as described below (Cluster validation, Tables S1-S3).

Descriptive statistical analysis was used to investigate the study cohort. Variable distribution was determined using the Kolmogorov-Smirnov test. Variables with non-Gaussian abnormal distribution were expressed by median and interquartile range (IQR). To test the quality of means of several distributions, the Kruskal Wallis test was used. For categorical data, chi-squared or Fisher's exact tests were used. The Bonferroni correction was performed for multiple comparisons to avoid the risk of type 1 errors.

Multivariate analysis was assessed by the logistic regression to test factors associated with TDRM by drug class and was based on unstandardized effect-size statistics. All statistically significant ($p<0.1$) univariate predictors of TDRM (sex, age at diagnosis (<50, ≥50), country of birth (SSA, EEU/CA, Israel, WCEU/NA), risk group (MSM, heterosexual contacts, PWID) and HIV-1 subtypes (B, A1, A6, C, other/recombinants) were all considered in multivariate analysis. Potential interactions were controlled by stratification on effect-measure-modifiers to assess heterogeneity of a measure across the levels of another factor. Predicted values of mean of response of logistic regression were preserved. Subsequently, TDRM probabilities (0.0-1.0) were visualized using R studio by multidimensional mapping. All statistical analysis was performed using IBM SPSS statistics 20 version and R studio version 1.2.1335.

Categorization of birthplace

Birthplace was categorized as follows which was based on https://aidsinfo.unaids.org/ definition: SSA (Ethiopia, Eritrea, Ghana, Sudan, South Africa ,Kenya), Eastern Europe and Central Asia, EEU/CA, (Russian Federation, Ukraine, Republic of Moldova, Belarus, Armenia, Azerbaijan, Tadzhikistan, Kyrgyzstan, Uzbekistan, Georgia), Western and Central Europe and North America, WCEU/NA (Austria, Belgium, France, Germany, Ireland, Sweden ,United Kingdom, Netherlands, Italy, Poland, Romania, Bulgaria, Hungary, Serbia, Turkey, United States), Other (Morocco, Algeria, Tunisia, Egypt, Iran, Iraq, Kuwait, United Arab Emirates, Costa Rica, Peru, Brazil, Afghanistan, India, Thailand, Colombia, Chile, Australia, Philippine, Mexico, Yemen, Venezuela, Argentina, Taiwan, Uruguay) and Israel.

Classification of sub-subtypes A1 and A6

To classify A subtypes into A1 and A6, the distribution of the pairwise distances between A6_pol_reference consensus, A6-POL-LA dataset, and the A1 reference sequences from Africa AB253421 (Rwanda) and AF004885 (Kenya) was measured. A6_pol_reference consensus (3) was generated using MutExt software (E.Schülter, University of Cologne) and the Advanced Consensus Maker tool from Los Alamos. A6-POL-LA dataset (3) included accession numbers of sequences from GenBank: EF545108 (Russia), EU861977 (Italy), AF193275 (Belarus), AF413987 (Ukraine). Distribution of pairwise distances was performed by Mega X (1) software version 10.1.1 . Table 1 below depicts the reference sequences used for this analysis.

Cluster validation

Clusters inferred from phylogenetic analysis were validated by repeating the analysis for subtype A, subtype B and subtype C with additional closely related reference sequences (Supplementary Table 3), as follows. A representative sequence was chosen from each cluster and the 10 most closely related sequences to the representative sequence were obtained using the Blastn sequence analysis tool (https://www.HIV-1.lanl.gov/content/sequence/BASIC_BLAST/basic_blast.html)(4), and added as control references (after removing the duplicate sequences). Overall, 119 unique sequences were chosen to validate the clusters (Supplementary Table S3). Drug resistance codons were

stripped from the new references to remove biases and all sequences for each subtype were aligned using Clustal Omega. Then phylogenetic analysis was repeated for A, B, C PR/RT sequences and Maximum likelihood phylogenetic trees were constructed using MEGAX (GTR+G+I model) with branch support assessed from 1000 bootstrap and visualized again. Bootstrap values ≥ 70 were considered significant.

Phylogenetic analysis was visualized using FigTree (http://tree.bio.ed.ac.uk/software/figtree).

Supplementary Table S1: Reference sequences used in the phylogenetic analysis.

Subtype	GenBank ref#	Country	Year
A1	AF004885	Kenya	1994
A1	AB253421	Rwanda	1992
A6 *pol* consensus	A6-POL-LA dataset		
A6	EF545108	Russia	2007
A6	EU861977	Italy	2008
A6	AF193275	Belarus	1999
A6	AF413987	Ukraine	2003
B	AY173951	Thailand	1990
C	AY772699	South Africa: Durban	2004

Supplementary Table S2: Number and length (bp) of sequences that were included in the phylogenetic trees.

	HIV-1 subtypes		
	**A**	**B**	**C**
**Number of sequencies**	482	732	313
**Alignment length**	826	833	900

Subtype C			
**C Cluster 1** North Israel	**C Cluster 2** Other	**C Cluster 3** additional	**C Cluster 4** clusters
**Representative: 56843,69662**	**Representative: 78873,67083**	**Representative: 85894,1000302**	**Representative: 85304,59421**
C.AT. KU574296	C.AT. KU574306	C.AT. KU574373	C.AT. KU574426
C.AT. KU574301	C.AT. KU574532	C.AT. KU574452	C.GB.2008.KU498602
C.AT. KU574391	C.CG.2006. KP889083	C.AT. KU574472	C.ZA.2009. KT032008
C.AT. KU574433	C.GB.2007.KU499113	C.BR.2009.KT746965	C.ZA.2009. KT032010
C.AT. KU574441	C.GB.2007.KU499180	C.GB.2008.KU498735	
C.AT. KU574448	C.GB.2008.KU498846	C.SE.1998.AF396682	
C.ZA.2008. KT032000	C.ZA.2008. KT032004	C.ZA.2008.KT032007	
C.ZA.2010. KT032021			

Supplementary Table S3: Additional Control reference sequences for phylogenetic analysis cluster validation

Accession numbers for reference sequences most closely related to a representative sequence in each of the clusters in subtypes A, B and C are listed. Representative sequences for each cluster (e.g. 1000403, 1000325) are highlighted in green shade. Sequences were located via BLAST program:
(https://www.hiv.lanl.gov/content/sequence/BASIC_BLAST/basic_blast.html).

Subtype A	
**A1 Cluster 1**	**A6 Cluster 2**
**Representative : 1000403,1000325**	**Representative : 83992,1000569**
A1.AT. KU574351	A6.AT. KU574304
A1.AT. KU574371	A6.AT. KU574406
A1.AT. KU574399	A6.AT. KU574408
A1.AT. KU574416	A6.AT. KU574435
A1. CG. 2006. KP889067	A6.AT. KU574447
A1. CG. 2006. KP889095	A6.AT. KU574474
A1. GB. 2007. KU498360	A6.AT. KU574479
A1. GB. 2007. KU498444	A6.AT. KU574480
A1. GB. 2008. KU498382	A6.AT. KU574512
A1. GR. 2004.GQ399093	A6. PL. 2000. KM057339

Subtype B				
**B Cluster 1**	**B Cluster 2**	**B Cluster 3**	**B Cluster 4**	**B Cluster 5**
**Representative: 1000438,87274, 62828**	**Representative: 1000369, 1000443**	**Representative: 1000425, 68738**	**Representative: 1000661, 73960**	**Representative: 84576, 1000631**
B.IL.2007.KM984944	B.IL.2005.KM985286	B.IL.2005.KM984931	B.IL.2008.KM985160	B.IL.2008.KM984988
B.IL.2007.KM985118	B.IL.2006.KM985283	B.IL.2009.KM985002	B.IL.2008.KM985216	B.IL.2009.KM984936
B.IL.2008.KM984993	B.IL.2007.KM985078	B.IL.2009.KM985238	B.IL.2008.KM985220	B.IL.2009.KM985032
B.IL.2008.KM985037	B.IL.2009.KM985021	B.IL.2009.KM985342	B.IL.2009.KM984975	B.IL.2009.KM985054
B.IL.2008.KM985282	B.IL.2009.KM985146	B.IL.2012.KM985052	B.IL.2009.KM985072	B.IL.2009.KM985113
B.IL.2009.KM985024	B.IL.2009.KM985321	B.IL.2012.KM985228	B.IL.2010.KM985317	B.IL.2009.KM985142
B.IL.2009.KM985105	B.IL.2010.KM985074	B.IL.2012.KM985275	B.IL.2011.KM985340	B.IL.2009.KM985206
B.IL.2009.KM985256	B.IL.2011.KM984926	B.IL.2012.KM985315	B.IL.2013.KM984923	B.IL.2010.KM984978
B.IL.2009.KM985270	B.IL.2011.KM984995	B.IL.2012.KM985323	B.IL.2013.KM984958	B.IL.2010.KM985025

B.IL.2009.KM985307	B.IL.2012.KM984899	B.IL.2013.KM985234	B.IL.2014.KM985144	B.IL.2011.KM985316
B.IL.2010.KM985007	B.IL.2012.KM985143	B.IL.2013.KM985269		B.IL.2012.KM985200
B.IL.2010.KM985071	B.IL.2012.KM985332	B.IL.2014.KM985313		B.IL.2013.KM985221
B.IL.2011.KM984984	B.IL.2012.KM985345			
B.IL.2011.KM985188	B.IL.2013.KM985257			
B.IL.2011.KM985211				
B.IL.2011.KM985291				
B.IL.2012.KM985035				
B.IL.2012.KM985258				
B.IL.2012.KM985325				
B.IL.2013.KM984933				
B.IL.2013.KM985039				
B.IL.2013.KM985084				
B.IL.2013.KM985111				
B.IL.2014.KM985309				

Bibliography

1. Kumar S, Stecher G, Li M, Knyaz C, Tamura K. MEGA X: Molecular evolutionary genetics analysis across computing platforms. Mol Biol Evol. 2018 Jun 1;35(6):1547–1549.
2. Posada D. jModelTest: phylogenetic model averaging. Mol Biol Evol. 2008 Jul;25(7):1253–1256.
3. Schlösser M, Kartashev VV, Mikkola VH, Shemshura A, Saukhat S, Kolpakov D, et al. HIV-1 Sub-Subtype A6: Settings for Normalised Identification and Molecular Epidemiology in the Southern Federal District, Russia. Viruses. 2020 Apr 22;12(4).
4. Altschul SF, Madden TL, Schäffer AA, Zhang J, Zhang Z, Miller W, et al. Gapped BLAST and PSI-BLAST: a new generation of protein database search programs. Nucleic Acids Res. 1997 Sep 1;25(17):3389–3402.

Supplementary Table S4: Proportion of most frequently detected TDRM and HIVdrm in newly diagnosed people living with HIV-1 (PLHIV) in Israel 2010-2018.

Drug class	TDRM	HIV-1 Subtype (N) All N= 1905	A1 46	A6 436	B 841	C 318	G AG 134	AE, A/AE 43	other 87	A1 vs A6	A1 vs B	A6 vs B	A1 vs C	A6 vs C	B vs C	A1 vs G AG	A6 vs G AG	A1 vs AE, A/AE	A6 vs AE, A/AE	A1 vs other	A6 vs other	B vs G AG	B vs AE, A/AE	B vs other	C vs G AG	C vs other
PI, n (%)	L10F	12 (0.6)		4 (0.9)	4 (0.5)	1 (0.3)	2 (1.5)		*1 (1.1)*			*0.394*		*0.311*	*0.648*		*0.550*				*0.860*	*0.177*		*0.474*	*0.149*	*0.335*
	K20T	2 (0.1)				1 (0.3)		*1 (2.3)*																		
	L23I	1 (0.1)					1 (0.7)																			
	L24F	1 (0.1)				1 (0.3)																				
	D30N	3 (0.2)			1 (0.1)	1 (0.3)	1 (0.7)								*0.456*							*0.144*			*0.550*	
	L33F	8 (0.4)	3 (6.5)	2 (0.5)	2 (0.2)				*1 (1.1)*	*< 0.001*	*< 0.001*	*0.355*								*0.082*	*0.509*			*0.134*		
	K43T	4 (0.2)		2 (0.5)			1 (0.7)		*1 (1.1)*								*0.784*				*0.509*					
	M46IL	18 (0.9)		2 (0.5)	4 (0.5)	6 (1.9)	3 (2.2)		*3 (3.4)*			*1.000*		*0.068*	*0.029*		*0.069*				*0.012*	*0.037*		*0.004*	*0.835*	*0.401*
	I47AV	4 (0.2)		4 (0.9)																						
	F53L	5 (0.3)		2 (0.5)	3 (0.4)							*0.797*														
	Q58E	15 (0.8)		1 (0.2)	13 (1.5)				*1 (1.1)*			*0.031*									*0.195*			*0.768*		
	V82AMST	3 (0.2)			1 (0.1)		1 (0.7)		*1 (1.1)*													*0.131*		*0.044*		
	N88D	1 (0.1)			1 (0.1)																					
	L89V	1 (0.1)		1 (0.2)																						
	L90M	39 (2)		2 (0.5)	34 (4)	1 (0.3)	1 (0.7)		*1 (1.1)*			*< 0.001*		*0.647*	*0.001*		*0.784*				*0.509*	*0.055*			*0.548*	
NRTI, n (%)	M41L	12 (0.6)		3 (0.7)	5 (0.6)	3 (0.9)	1 (0.7)					*0.831*		*0.456*	*0.525*		*1.000*					*0.891*			*0.832*	
	A62V	51 (2.7)		49 (11)		2 (0.6)								*< 0.001*												
	K65NR	2 (0.1)			1 (0.1)	1 (0.3)									*0.44*											
	D67EGN	12 (0.6)		6 (1.4)	4 (0.5)	2 (0.6)						*0.100*		*0.290*	*0.834*											
	T69D	2 (0.1)			2 (0.2)																					
Drug class	TDRM	All N= 1905	A1 46	A6 436	B 841	C 318	G AG 134	AE, A/AE 43	other 87	A1 vs A6	A1 vs B	A6 vs B	A1 vs C	A6 vs C	B vs C	A1 vs G AG	A6 vs G AG	A1 vs AE, A/AE	A6 vs AE, A/AE	A1 vs other	A6 vs other	B vs G AG	B vs AE, A/AE	B vs other	C vs G AG	C vs other
	K70ENRT	7 (0.4)	1 (2.2)	3 (0.7)	1 (0.1)	1 (0.3)	1 (0.7)			*0.291*	*0.002*	*0.065*	*0.1*	*0.456*	*0.44*	*0.398*	*1.000*					*0.131*			*0.548*	
	L74V	2 (0.1)				1 (0.3)			*1 (1.1)*																	*0.335*
	V75I	2 (0.1)		1 (0.2)	1 (0.1)							*0.644*														
	F77L	2 (0.1)			1 (0.1)			*1 (2.3)*															*0.003*			
	Y115F	1 (0.1)					1 (0.7)																			
	M184IV	18 (0.9)		7 (1.6)	5 (0.6)	6(1.9)						*0.079*		*0.755*	*0.043*											
	L210W	6 (0.3)		3 (0.7)	2 (0.2)	1 (0.3)						*0.163*		*0.456*	*0.750*											

	**T215ADEFI**LNS**Y**	34 (1.8)	1 (2.2)	5 (1.1)	19 (2.3)	6 (1.9)	2 (1.5)		*1 (1.1)*	*0.516*	*0.965*	*0.136*	*0.890*	*0.362*	*0.678*	*0.751*	*0.8*			*0.619*	*1.000*	*0.557*		*0.467*	*0.769*	*0.612*
	**K219EQR**	6 (0.3)		3 (0.7)	2 (0.2)		1 (0.7)					*0.163*					*1.000*					*0.478*				
**NNRTI, n (%)**	A98G	14 (0.7)		2 (0.5)	3 (0.4)	8 (2.5)			*1 (1.1)*			*0.797*		*0.019*	*0.001*						*0.509*			*0.362*		*0.431*
	**K101E**	3 (0.2)			1 (0.1)	1 (0.3)		*1 (2.3)*							*0.456*								*0.003*			
	**K103NS**	94 (4.9)	22 (48)	5 (1.1)	54 (6.4)	7 (2.2)	3 (2.2)		*3 (3.4)*	*< 0.001*	*< 0.001*	*0.004*	*< 0.001*	*0.230*	*0.009*	*< 0.001*	*0.337*			*< 0.001*	*0.105*	*0.078*		*0.337*	*1.000*	*0.522*
	**K103N**	86 (4.5)	16 (35)	5 (1.1)	53 (6.3)	7 (2.2)	2 (1.5)		*3 (3.4)*	*< 0.001*	*< 0.001*	*< 0.001*	*< 0.001*	*0.230*	*0.005*	*< 0.001*	*0.710*			*< 0.001*	*0.105*	*0.025*		*0.280*	*0.627*	*0.522*
	**K103S**	8 (0.4)	6 (13)		1 (0.1)		1 (0.7)				*< 0.001*					*< 0.001*						*0.131*				
	**V106AM**	4 (0.2)		2 (0.5)		2 (0.6)								*0.854*												
	V108I	5 (0.3)		2 (0.5)	3 (0.4)							*0.797*														
	E138AGKQ	109 (5.7)	25(54)	23 (5.3)	25 (3)	13 (4.1)	19 (14)	*1 (2.3)*	*3 (3.4)*	*< 0.001*	*< 0.001*	*0.041*	*< 0.001*	*0.447*	*0.350*	*< 0.001*	*< 0.001*	*< 0.001*	*0.391*	*< 0.001*	*0.458*	*< 0.001*	*0.792*	*0.836*	*< 0.001*	*0.767*
	E138A	70 (3.7)	1 (2.2)	17 (3.9)	21 (2.5)	10 (3.1)	17 (12.7)	*1 (2.3)*	*3 (3.4)*	*0.564*	*0.900*	*0.163*	*0.738*	*0.559*	*0.572*	*0.041*	*< 0.001*	*0.975*	*0.599*	*0.700*	*0.824*	*< 0.001*	*0.572*	*0.615*	*< 0.001*	*0.888*
	E138G	10 (0.5)	2 (4.3)	4 (0.9)	1 (0.1)	2 (0.6)	1 (0.7)			*0.046*	*< 0.001*	*0.026*	*0.023*	*0.642*	*0.119*	*0.096*	*0.826*					*0.131*			*0.902*	
	E138K	3 (0.2)		1 (0.2)	1 (0.1)	1 (0.3)						*0.644*		*0.783*	*0.440*											
	E138Q	26 (1.4)	22 (48)	1 (0.2)	2 (0.2)		1 (0.7)			*< 0.001*	*< 0.001*	*1.000*				*< 0.001*	*0.369*					*0.299*				
	V179DEL	22 (1.1)		7 (1.6)	7 (0.8)	3 (0.9)	4 (3)		*1 (1.1)*			*0.189*		*0.403*	*0.867*		*0.303*				*0.728*	*0.024*		*0.769*	*0.096*	*0.864*
	**Y181CI**	11 (0.6)		3 (0.7)	3 (0.4)	5 (1.6)						*0.472*		*0.238*	*0.039*											
	**Y188CL**	2 (0.1)		1 (0.2)		1 (0.3)								*0.783*												
	**G190AS**	7 (0.4)		1 (0.2)		5 (1.6)	1 (0.7)							*0.032*			*0.369*								*0.447*	
	H221Y	3 (0.2)			3 (0.4)																					
	**P225H**	1 (0.1)	1 (2.2)																							
	K238NT	4 (0.2)		2 (0.5)		2 (0.6)								*0.854*												
**INSTI, n (%)**		**N= 479**	**A1 8**	**A6 112**	**B 202**	**C 65**	**G AG 48**	**AE, A/AE 10**	**other 34**	**A1 vs A6**	**A1 vs B**	**A6 vs B**	**A1 vs C**	**A6 vs C**	**B vs C**	**A1 vs G AG**	**A6 vs G AG**	**A1 vs AE, A/AE**	**A6 vs AE, A/AE**	**A1 vs other**	**A6 vs other**	**B vs G AG**	**B vs AE, A/AE**	**B vs other**	**C vs G AG**	**C vs other**
	L74I	44 (9.2)		38 (34)	3 (1.5)	1 (1.5)	2 (4.2)					*< 0.001*		*< 0.001*	*1.000*		*< 0.001*					*0.233*			*0.380*	
	L74V	3 (0.6)	3 (38)																							
	T97A	7 (1.5)		1 (0.9)	2 (1)	2 (3.1)	2 (4.2)					*0.931*		*0.278*	*0.228*		*0.161*					*0.115*			*0.756*	
	E157Q	6 (1.3)			3 (1.5)	1 (1.5)	1 (2.1)		*1 (2.9)*						*1.000*							*0.767*		*0.560*	*0.811*	*0.637*
	G163KR	3 (0.6)							*3 (8.8)*																	

TDRM, transmitted drug resistance mutations are highlighted in bold blue; HIVdrm- HIV-1 drug resistance mutations; NRTI, nucleoside reverse transcriptase inhibitors; NNRTI, non-nucleoside transcriptase inhibitors; PI, protease inhibitors; INSTI – integrase strand transfer inhibitors. Data are presented as n (%). Differences in proportions were measured using the chi-squared test. Empty cells, n=zero.

Supplementary Table S5: Interactions and main effects of variables related to TDRM

Supplementary S5a. Variables related to overall TDRM

Interactions				Main effects		
Variables	Coefficients	OR (95% CI)	*p*	Coefficients	OR (95% CI)	*p*
Age (<50) * HIV-1 subtype A1	2.457	11.671 (6.010-22.666)	<0.001			
Age (<50)				-0.310	0.733 (0.512-1.049)	0.090
HIV-1 subtype A1				2.350	10.489 (5.752-19.126)	<0.001
Age (≥50) * HIV-1 Subtype A1	2.139	8.488 (2.103-34.256)	0.003			
Age (≥50)				0.310	1.364 (0.953-1.952)	0.090
HIV-1 subtype A1				2.350	10.489 (5.752-19.126)	<0.001
Age (<50) * Sex (male)	0.534	1.705 (1.120-2.596)	0.013			
Age (<50)				-0.310	0.733 (0.512-1.049)	0.090
Sex (male)				0.335	1.397 (0.978-1.996)	0.066
Age (≥50) * Sex (male)	0.962	2.618 (1.281-5.349)	0.008			
Age (≥50)				0.310	1.364 (0.953-1.952)	0.090
Sex (male)				0.335	1.397 (0.978-1.996)	0.066
Age (<50) * Birthplace (IL)	0.728	2.071 (1.498-2.863)	<0.001			
Age (<50)				-0.310	0.733 (0.512-1.049)	0.090
Birthplace (IL)				0.476	1.610 (1.216-2.133)	0.001
Birthplace (EEU/CA) * Transmission mode (PWID)	-0.703	0.495 (0.270-0.909)	0.023			
Birthplace (EEU/CA)				-0.572	0.564 (0.393-0.810)	0.002
Transmission mode (PWID)				-0.629	0.533 (0.319-0.892)	0.017
Sex (Male) * HIV-1 subtype A1	2.598	13.431 (6.556-27.516)	<0.001			
Sex (male)				0.335	1.397 (0.978-1.996)	0.066
HIV-1 subtype A1				2.350	10.489 (5.752-19.126)	<0.001

Sex (female) * HIV-1 subtype A1	2.627	13.838 (2.983-64.194)	0.001			
Sex (female)				-0.334	0.716 (0.502-1.023)	0.067
HIV-1 subtype A1				2.350	10.489 (5.752-19.126)	<0.001
Birthplace (IL) * HIV-1 subtype A1	2.570	13.064 (6.571-25.972)	<0.001			
Birthplace (IL)				0.476	1.610 (1.216-2.133)	0.001
HIV-1 subtype A1				2.350	10.489 (5.752-19.126)	<0.001
Transmission mode (PWID) * HIV-1 subtype A6	-1.069	0.343 (0.172-0.683)	0.002			
Transmission mode (PWID)				-0.629	0.533 (0.319-0.892)	0.017
HIV-1 subtype A6				-0.635	0.530 (0.364-0.772)	0.001

Supplementary S5b. Variables related to NNRTI TDRM

Interactions				Main effects		
Variables	Coefficients	OR (95% CI)	*p*	Coefficients	OR (95% CI)	*p*
Birthplace (IL) * HIV-1 subtype A1	3.414	30.889 (14.717-62.752)	<0.001			
Birthplace (IL)				0.686	1.986 (1.357-2.905)	<0.001
HIV-1 Subtype A1				3.042	20.940 (11.331-38.700)	<0.001
Birthplace (EEU/CA) * HIV-1 subtype A6	-1.231	0.292 (0.141-0.606)	0.001			
Birthplace (EEU/CA)				-0.882	0.414 (0.242-0.707)	0.001
HIV-1 subtype A6				-0.080	0.340 (0.185-0.622)	<0.001
Transmission mode (PWID) * HIV-1 subtype A6	-2.654	0.070 (0.010-0.507)	0.008			
Transmission mode (PWID)				-1.069	0.343 (0.149-0.788)	0.012
HIV-1 subtype A6				-0.080	0.340 (0.185-0.622)	<0.001

Supplementary S5c. Variables related to PI TDRM

Interactions				Main effects		
Variables	Coefficients	OR (95% CI)	*p*	Coefficients	OR (95% CI)	*p*
Birthplace (IL) * Sex (male)	0.792	2.209 (1.027-4.753)	0.043			
Birthplace (IL)				0.482	1.619 (0.922-2.643)	0.054
Sex (male)				0.703	2.020 (0.995-4.101)	0.052
Birthplace (EEU/CA) * Transmission mode (sexual contacts)	-1.312	0.269 (0.083-0.872)	0.029			
Birthplace (EEU/CA)				-0.527	0.590 (0.315-1.108)	0.101
Transmission mode (sexual contacts)				-0.814	0.443 (0.241-0.815)	0.009
Transmission mode (sexual contacts) * HIV-1 subtype A6	-1.601	0.202 (0.049-0.835)	0.027			
Transmission mode (sexual contacts)				-0.814	0.443 (0.241-0.815)	0.009
HIV-1 subtype A6				-0.737	0.478 (0.236-0.970)	0.041

TDRM, surveillance drug resistance mutations; NNRTI, non-nucleoside reverse transcriptase inhibitors; PI, protease inhibitors. First, univariate analysis was performed for potential predictors of TDRM (sex, age at diagnosis (<50, >50); birthplace (SSA, EEU/CA, Israel, WCEU/NA; transmission mode -MSM, heterosexual contacts, PWID; HIV-1 subtypes -B, A1, A6, C, other/recombinants). All statistically significant (p<0.1) predictors were included into the multivariate logistic regression analysis. The resultant coefficients and Odds ratio (OR) of 95% CI of the interactions (interactions are marked by "*") and of the main effects, are displayed.

Supplementary Table S6: Proportion of most frequently detected DRM and patient demographics in the largest transmission clusters.

Subtype	Cluster number	# of sequences	Drug Class	Major DRM	Risk group, n/N	Birthplace, n/N
**A6**	1	68	PI	I47V 1/68	3/68 MSM;	1/68 SSA;
			NRTI	L210W 1/68	62/68 PWID;	46/68 EEU/CA;
			NNRTI	V179D 1/68	1/68 Hetero;	18/68 IL;
					2/68 Unknown;	3/68 Other;
**A1**	2	43	PI	L33F 3/43	28/43 MSM;	2/43EEU/CA;
			NRTI	K70N 1/43	4/43 PWID;	3/43 WCEU/NA;
				T215I 1/43	10/43 Hetero;	34/43 IL;
			NNRTI	K103N 15/43	1/43 Unknown;	4/43 Other;
				K103S 6/43		
				E138A 1/43		
				E138G 2/43		
				E138Q 22/43		
**B**	1	76	PI	M46I 1/76	63/76 MSM;	9/76 EEU/CA;
				L10F 1/76	7/76 PWID;	2/76 WCEU/NA;
			NRTI	F77L 1/76	4/76 Hetero;	62/76 IL;
			NNRTI	Y181C 3/76	2/76 Unknown;	3/76 Other;
				H221Y 3/76		
	2	52	PI	Q58E 11/52	47/52 MSM;	8/52 EEU/CA;
			NRTI	M184V 1/52	1/52 PWID;	2/52 WCEU/NA;
			NNRTI	V179D 1/52	4/52 Hetero;	42/52 IL;
	3	36	PI	M46I 1/36	23/36 MSM;	5/36 EEU/CA;
				L33F 1/36	3/36 PWID;	29/36 IL;
					10/36 Hetero;	2/36 Other;

Subtype	Cluster number	# of sequences	Drug Class	Major DRM	Risk group, n/N	Birthplace, n/N
	4	30	NNRTI	K103N 17/30	25/30 MSM;	1/30 SSA;
					2/30 PWID;	2/30 EEU/CA;
					3/30 Hetero;	1/30 WCEU/NA;
						24/30 IL;<br>2/30 Other;
	5	27	PI	L90M 25/27	22/27 MSM;	1/27 SSA;
			NRTI	V75I 1/27	3/27 Hetero;	2/27EEU/CA;
			NNRTI	E138Q 1/27	2/27 Unknown;	1/27 WCEU/NA;
						21/27 IL;
						2/27 Other;
**C**	1	21	NNRTI	K103N 1/21	12/21 MSM;	2/21EEU/CA;
					2/21 PWID;	16/21 IL;
					7/21 Hetero;	3/21 Other;

DRM, drug resistance mutations; NNRTI, non-nucleoside reverse transcriptase inhibitors; NRTI, nucleotide reverse transcriptase inhibitors; PI, protease inhibitors. Country of origin: SSA - Sub Saharan Africa; EEA/CA - Eastern Europe and Central Asia; WCEU/NA - Western and Central Europe and North America; IL- Israel; PWID - people who use injection drug; MSM – men who have sex with men; HETERO – heterosexual contacts.
